# Accurate prediction of candidate lncRNAs associated with DNA damage response based on gene expression patterns from graph neural networks

**DOI:** 10.1093/bioadv/vbag119

**Published:** 2026-04-26

**Authors:** Snehal Shah, Liangjiang Wang

**Affiliations:** Department of Genetics and Biochemistry, Clemson University, Clemson, SC 29631, USA; Center for Human Genetics, Clemson University, Greenwood, SC 29646, USA; Department of Genetics and Biochemistry, Clemson University, Clemson, SC 29631, USA; Center for Human Genetics, Clemson University, Greenwood, SC 29646, USA

## Abstract

**Motivation:**

DNA damage response (DDR) is essential for maintaining genome stability and preventing tumorigenesis. While protein-coding DDR genes have been extensively investigated, long non-coding RNAs (lncRNAs) remain relatively understudied despite the growing evidence of their involvement in DDR. Particularly, it is rather challenging to systematically identify DDR-associated lncRNAs through experimental approaches, which are often time-consuming, labor-intensive, and expensive. Moreover, lncRNAs lack translational open reading frames often targeted by experimental methods.

**Results:**

In this study, we have developed a new machine learning approach, GlncDDR, which utilizes graph-based node embedding of gene expression features and supervised learning algorithms to predict candidate lncRNAs associated with DDR. GlncDDR models achieved robust predictive performance with ROC-AUC reaching ∼0.93 on test data. We used the models to predict 1232 candidate lncRNAs, including several known DDR regulators such as *JADRR, PINCR, TP53TG1, HOTAIR, MALAT1, ENRICD, and DINOL*. Interestingly, 212 of the candidates were found to be located near known DDR genes in the genome, supporting the potential functions of these lncRNAs in DDR. The results demonstrate the effectiveness of predicting DDR-associated lncRNAs based on cancer transcriptomic data and provide valuable targets for exploring the non-coding regulatory landscape of genome stability and cancer drug discovery.

**Availability:**

The source code and datasets used in the study are available at https://github.com/BioDataLearning/GlncDDR.git

## 1 Introduction

In complex life, the genetic information encoded within the DNA determines the structure and function of cells. However, DNA is constantly under threat from various intrinsic and extrinsic factors such as ionizing radiations, ultraviolet radiations, reactive oxygen species (ROS), and oncogenic mutations resulting in DNA damages (Wu *et al*. 2018). Such damages, ranging from single-strand breaks to severe double-strand breaks, can cause mutations and chromosomal abnormalities, contributing to diseases like cancer. To maintain genomic stability and normal cell growth, cells rely on DNA damage response (DDR) pathways, which detect, signal, and repair DNA lesions.

Long non-coding RNAs (lncRNAs) play essential roles in various biological processes. Particularly, extensive research affirms the involvement of lncRNAs in all three DDR pathways: ataxia telangiectasia and Rad3-related protein (ATR), ataxia telangiectasia-mutated kinase (ATM), and DNA-dependent protein kinase (DNA-PK). Upon DNA damage, the ATM pathway triggers the production of an ATM-dependent lncRNA, *JADE*, which facilitates DNA repair by halting G1/S checkpoints, suppressing apoptosis, and recruiting Mdc1, a protein important for DDR (Wan *et al*. 2013). Previous studies also suggest that lncRNAs are involved in maintaining the telomere integrity and thus crucial for chromosomal stability (Aguado, d’Adda di Fagagna and Wolvetang 2020), promote DDR by interacting with ATR and ATM which are critical regulators for phosphorylating the p53 protein (Abuetabh *et al*. 2022). Although protein-coding DDR genes have been identified using experimental approaches, genome-wide identification of DDR-associated lncRNAs is still challenging. Unlike protein-coding genes, lncRNAs lack open reading frames (ORFs) to encode functional protein products, thus making lncRNAs hard to be disrupted by genome-editing techniques such as CRISPR-Cas9. Moreover, the functional regions of lncRNAs are often unclear, and lncRNA function may depend on the act of transcription rather than the transcript products (Goyal *et al*. 2017).

Given the abundance of genomic and transcriptomic data, computational methods offer a promising avenue for predicting candidate lncRNAs associated with DDR. However, conventional methods that rely solely on sequence features or structural similarity often overlook the complex interactions and functional relevance embedded in gene expression data (Huarte 2015, Liu *et al*. 2019). Transcriptomic data analysis can provide valuable insights into active gene expression levels, latent expression patterns, functional relationships between genes, regulatory mechanisms, and alternative splicing events. Nevertheless, the high dimensionality of gene expression data may introduce challenges, such as model overfitting, when directly used by computational methods (Clarke *et al*. 2008). Recently, gene set variation analysis (GSVA) has been used to assess DDR enrichment scores in expression data, and differential expression analysis has identified lncRNAs linked to DDR pathway activity differences between groups with varying lncRNA expression levels (Liu *et al*. 2024). Moreover, least absolute shrinkage and selection operator-Cox regression analysis has been applied to hepatocellular carcinoma gene expression data from TCGA to identify lncRNAs associated with DDR and predict prognosis and immunotherapy response (Huang *et al*. 2024). However, the complex relationships between lncRNAs and DDR pathways might not be fully modeled in these two studies. GSVA relies on pre-ranked genes, potentially limiting the identification of novel lncRNAs. The other study (Huang *et al*. 2024) employed a statistical method that captured a linear relationship between gene expression and overall survival, which may not accurately reflect the non-linear and complex interactions between lncRNAs and DDR pathways. New approaches are needed to accurately predict DDR-associated lncRNAs without solely relying on pre-ranked genes or linear models.

Biological entities such as genes and proteins form interconnected networks, where relationships can reveal functional interactions (Oulas *et al*. 2019). These networks can be used to identify key molecular players and their functional connections in biological processes (Li and Zhang 2017). The notion of biological networks states that diseases with similar phenotypic characteristics are driven by functionally related genes that are spatially proximate (Wang *et al*.2011). Recently, deep learning techniques have been applied to unstructured or non-Euclidean data structures such as graphs, enabling more advanced modeling of complex structures (Bacciu *et al*. 2020). Particularly, graph neural networks (GNNs) have emerged as a powerful tool for analyzing graph-structured data. GNNs take non-Euclidean data graphs as input features and utilize message passing between nodes to capture topological, scale, and heterogeneity information (Chatzianastasis *et al*. 2023). In biological networks, nodes represent entities such as genes and proteins, while edges model the relationships or interactions between nodes (Zhang *et al*. 2021). GNN tasks can be categorized into node classification, link prediction, and whole-graph classification. Node classification can be performed using node embeddings for feature representation learning or by applying graph convolutional networks (GCNs) directly to the graph structure. This flexibility allows GNNs to offer a comprehensive way to study complex biological systems. The inherent ability of GNN node embedding to model high-dimensional, non-linear interactions between biological entities offers a significant advantage over traditional linear models, making them particularly useful for understanding complex processes such as those involved in gene regulation and disease pathways (Grover and Leskovec 2016).

In this study, we have developed GlncDDR (Graph neural network-based prediction of lncRNAs associated with DDR) to accurately predict DDR-associated lncRNAs using gene expression patterns. Transcriptomic data from The Cancer Genome Atlas (TCGA) were used as the large-scale expression measurements of both coding and non-coding genes in various cancer cells. The TCGA dataset is particularly relevant as the perturbed cellular processes in cancer enable the representation learning of complex regulatory networks involved in DDR. Notably, GlncDDR predicted *JADRR, PINCR, TP53TG1, HOTAIR, MALAT1, ENRICD*, and *DINOL*, which are already known to be associated with DDR, along with other candidate lncRNAs such as *POT1-AS1*, *WAC-AS1*, *LINC02381*, *IRF1-AS1*, and *C8orf8*6. These lncRNAs may be involved in cell proliferation, apoptosis, and cell migration, acting as tumor suppressors. Thus, our study has demonstrated accurate prediction of candidate lncRNAs associated with DDR using node2vec as a feature representation technique. The candidate lncRNAs predicted by GlncDDR may provide helpful information for further experimental validation, drug discovery, and cancer therapy.

## 2 Methods

**Figure 1:**
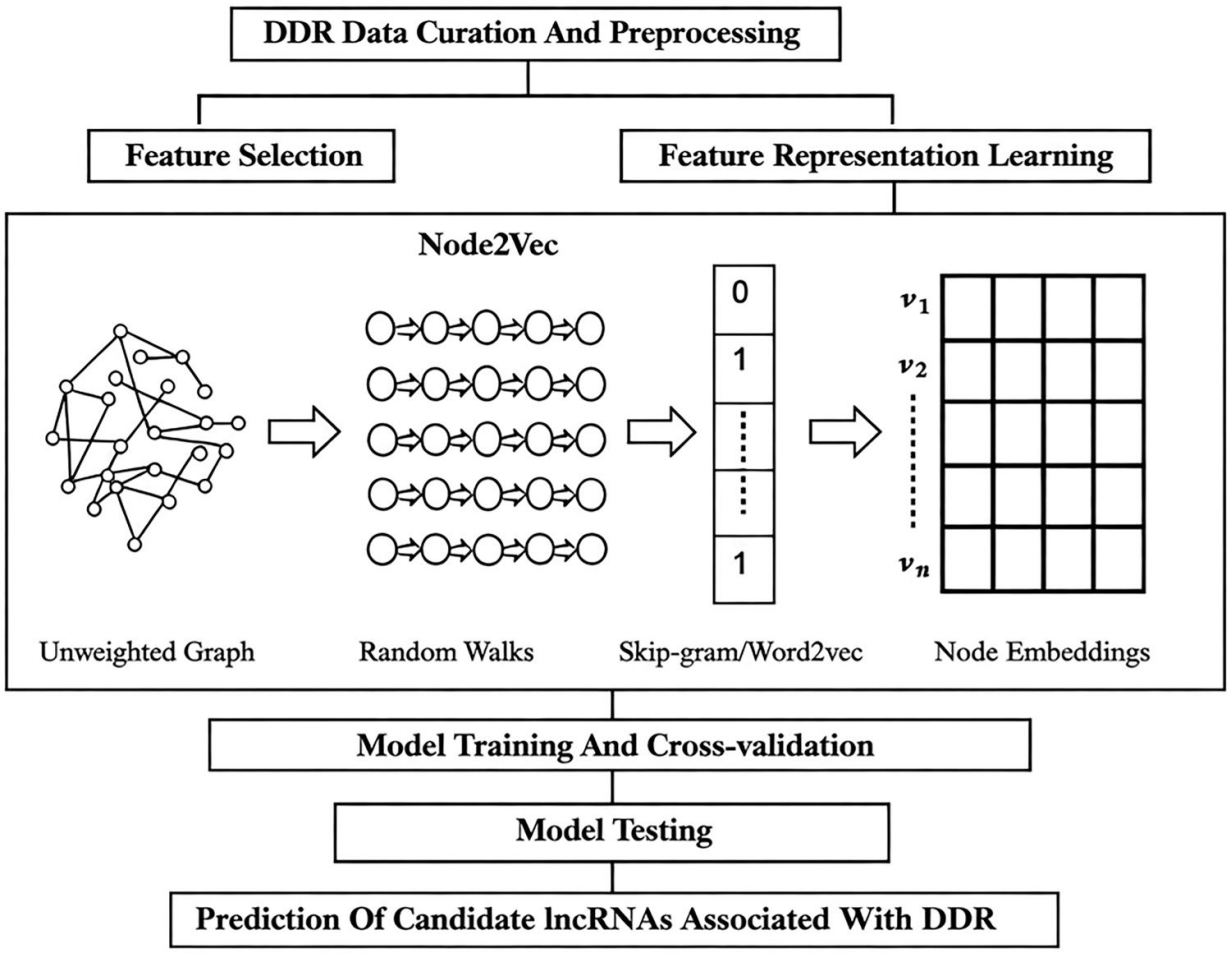
Schematic diagram of GlncDDR model construction. First, DDR and non-DDR genes were collected, followed by data pre-processing. Second, different dimensionality reduction techniques were examined for feature selection and representation learning. Lastly, various machine learning algorithms were used for model training and cross-validation. The models were further tested on an independent dataset and then used to predict candidate lncRNAs associated with DDR.

In this study, we aimed to predict candidate lncRNAs associated with DDR in cancer cells based on gene expression patterns (Fig. 1). First, DDR genes (positive instances) and non-DDR genes (negative instances) were compiled from published studies (Pearl *et al*. 2015, Knijnenburg *et al*. 2018, Weir *et al*. 2022). Second, we used node2vec for feature representation learning to uncover topological structures and pertinent biological information encoded within each gene’s high-dimensional expression data. Among several graph-based embedding techniques (Hou *et al*. 2020, Makarov *et al*. 2021), we chose node2vec to reduce the high dimensionality of the TCGA transcriptomic data. Node2vec employs a biased random walk mechanism to balance homophily and structural equivalence, thereby capturing both local and global topological structures within networks (Grover and Leskovec 2016). As a semi-supervised network embedding algorithm, node2vec is also well-suited for biological applications with limited labelled data. Lastly, different machine learning algorithms were used for model construction with the learned features to perform binary classification of DDR genes in cancer cells.

### 2.1 Curation of datasets for model construction

For GlncDDR model training and cross-validation, we curated a dataset comprising of 491 DDR genes (positive instances) and 1048 non-DDR genes (negative instances). The positive instances consisted of protein-coding genes integral to the DDR pathways, and were sourced from published studies (Pearl *et al*. 2015, Knijnenburg *et al*. 2018, Weir *et al*. 2022). The negative instances comprised protein-coding genes lacking experimental evidence for DDR involvement, and were also sourced from a previous study (Weir *et al*. 2022). Genes with ambiguous annotations were excluded from both positive and negative instances to avoid potential label contamination. To further evaluate model performance, we curated a separate test dataset using genes from additional DDR studies (Zimmermann *et al*. 2018, Hustedt *et al*. 2019, Olivieri *et al*. 2020). The test dataset comprised of 312 DDR genes and 8266 non-DDR genes. To avoid data leakage, gene-level filtering was applied using Ensembl IDs to ensure that all the DDR genes and non-DDR genes in the test dataset were not included in the training dataset. The additional non-DDR genes were compiled from OMIM (https://www.omim.org). We applied filtering criteria to avoid genes linked to DDR pathways or DDR-related cancer genes. The filtering terms such as 'sarcoma’, 'lymphoma’, and 'cancer’ were applied. Moreover, we tested the performance of GlncDDR on protein-coding genes downloaded from GENCODE Release 36 (GRCh38.p13), excluding those previously used in training and testing, resulting in 8436 protein-coding genes. Finally, to predict candidate lncRNAs associated with DDR, we utilized a comprehensive list of lncRNAs from GENCODE Release 36 (https://www.gencodegenes.org/human/release_36.html).

### 2.2 Use of TCGA transcriptomic data as features

This study utilized RNA-seq data from The Cancer Genome Atlas (TCGA) to construct machine learning models for DDR gene prediction. TCGA provides comprehensive gene expression profiles of 60,660 genes in 11,273 patients of 33 different cancer types (https://portal.gdc.cancer.gov/). The transcriptomic data in Fragment Per kilobase of transcripts per Million map reads (FPKM) units were processed to avoid sparsity in the data because of potential biases and differences in the sequencing depth and gene length. The FPKM values below 1 were set to 1, followed by log_2_ transformation. Then, genes with log_2_FPKM values of zero in over 75% of the samples were removed to ensure dataset quality. Finally, we normalized the data using the min-max scaling technique embedded in the scikit-learn (v1.3) Python library.

### 2.3 Gene representation learning

To extract relevant information from the gene expression data, avoid the risks of model overfitting, and ultimately enhance the model’s ability of generalization, feature representation learning was performed. We examined two different representation learning methods, node2vec embeddings and autoencoder, and compared their effectiveness with that of conventional feature selection to improve model performance.

Node2vec is a semi-supervised network embedding algorithm that transforms networks into numerical representations (Grover and Leskovec 2016). In this study, the input network for node2vec was constructed using Weighted Gene Co-expression Network Analysis (WGCNA) (Langfelder and Horvath 2008). We used the Python package, PyWGCNA, to perform WGCNA (Rezaie, Reese, and Mortazavi 2023). The soft-threshold power was estimated using the pickSoftThreshold function of the PyWGCNA package and the scale-free topology fit index ($R^{2}$) was estimated automatically by the pickSoftThreshold function. The resulting *β* value was used to compute the adjacency matrix, transforming pairwise Pearson correlations between genes into weighted connections. The adjacency matrix was then used to construct an undirected gene network using the NetworkX package (version 3.2.1), with nodes representing genes and edge weights corresponding to co-expression signals. Node2vec took the co-expression network as input to generate the embeddings by performing biased random walks, balancing local (Breadth‐First Search, BFS) and global (Depth-First Search, DFS) traversals to capture diverse neighborhood information. Specifically, for a walk of length l, let the walk sequence be $c_{0},c_{1},\ldots ,\;c_{l}$. At each step i (for i≥1), the probability of transitioning from current node $v=c_{i-1}\;$to a candidate neighbor x is defined as:


(1) P(ci=x|ci−1=v)= {πvxZ if (v,x)∈E0 otherwise 

Where $\pi_{vx}$ is the unnormalized transition probability between node *v* and *x* and *Z* is a normalized constant summing over all neighbors u of v. To ensure that the random walk can emphasize either local neighborhoods (BFS) or explore farther away (DFS), node2vec introduces two hyperparameters, return parameter *p* and in-out parameter *q.* Specifically, if $t=c_{i-2}$ is the previous node and x is a candidate next node, define the shortest path as $d_{t,x}=\mathrm{distance}(t,x)\;\in \{0,1,2\}$. Then calculating the unnormalized transitional probabilities $\pi_{vx}$ on edges (v,x) leading from v is set to $\pi_{vx}=\alpha_{pq}(t,\;x)\cdot \;w_{vx}$, where $w_{vx}$ is the (possibly weighted) edge between v and x, and


(2) αpq(t,x) = {1 p if Dtx=01 if Dtx=11q if Dtx=2 

Where $D_{tx}$ represents the shortest path distance between nodes t and x. The $D_{tx}$ should be between {0, 1, 2}; thus, two parameters are sufficient to guide the walk. The whole procedure of computing probabilities and performing second-order biased random walks is iteratively executed until a predefined length of walk is attained. Finally, inspired by the skip-gram model, which is used in natural language processing to learn continuous feature representation for words by optimizing the likelihood objective using stochastic gradient descent (SGD) with negative sampling, we use skip-gram for network representations. Nodes are words, and sentences are the connections between the nodes stored as sequences of random walks. These sequences of random walks are fed into skip-gram with a negative sampling model, which retrieves the hidden layer weights as node embeddings. In this study, the node2vec analysis was performed in Python (v3.10) with the default hyperparameter settings (*p *= 1 and *q *= 1), which were shown to perform robustly for biological network embedding tasks (Grover and Leskovec 2016), and the walk length *l *= 10. We tested embedding vectors of different dimension sizes (10, 20, 30, 50, 100, 150, 200, 250, 300) and selected the optimal dimension size based on model performance. Additionally, post hoc interpretability analysis was conducted using SHAP to quantify the contributions of embedding-level features to model predictions.

An autoencoder is an unsupervised neural network that learns to efficiently compress and encode data before reconstructing it to closely match the original input. It consists of an input layer, an output layer, and one or more hidden layers that form the encoder and decoder components. The encoder compresses input data into a low-dimensional representation known as the code, while the decoder reconstructs this code to produce an output that resembles the initial input. In this study, we utilized a basic autoencoder with a single hidden layer (*x_code_*) positioned between the encoder and decoder. Both the encoder and decoder employed the rectified linear unit (ReLU) as the nonlinear activation function. Mean squared error acted as the loss function. We developed the autoencoder using TensorFlow 2.0 (https://github.com/keras-team/keras), a Python library. The training process included 45 epochs and a batch size of 64. To prevent overfitting and maintain the optimal neural network, we implemented early stopping and checkpoints to monitor validation loss. We tested *x_code_* dimensionality at different vector sizes of 10, 20, 30, 50, 150, 200, 250, and 300.

### 2.4 Model training

To accurately predict candidate lncRNAs associated with DDR, machine learning algorithms such as logistic regression (LR), random forest (RF), and support vector machine (SVM) were used for model construction. These classical learning algorithms were chosen, considering the relatively small size of training dataset available and the very high dimensionality of the transcriptomic data, whereas deep learning models normally require substantially large datasets to achieve robust performance and avoid overfitting. LR is a statistical algorithm that uses a logistic or sigmoid function to predict the relationship between outcomes and independent variables. RF is an ensemble method that constructs multiple decision trees on bootstrap sample data with a randomly selected subset of features considered at each node split, with final prediction aggregating outputs across all trees (Breiman 2001). This combination of bagging and random feature selection reduces overfitting and variance, making RF robust for high dimensional classification. SVM is a supervised machine learning algorithm that identifies an optimal hyperplane (decision boundary) in high-dimensional space to classify the target labels distinctly. The hyperplane is optimal when the distance between two data points of the target labels is maximum. SVM is a commonly used algorithm in machine learning as it helps classify data even when they are not linearly separable (Cortes and Vapnik 1995). The kernel function of SVM helps transform the data into two categories of our target labels. The commonly used kernel function for non-linear data is the radial basis function (RBF). The kernel function has a regularization parameter called C, which controls misclassification while maximizing the hyperplane, resulting in overfitting. Another critical parameter while using the RBF kernel is gamma, which decides the width of the kernel function and the distance of a single data point. In this study, SVM models were constructed using the SVC function of the scikit-learn Python library (Pedregosa *et al*. 2011).

### 2.5 Model performance evaluation

The performance of GlncDDR was evaluated by five-fold cross-validation. Considering the imbalanced dataset, the following performance metrics were used:


(3) $$\mathrm{Accuracy}=\;\frac{TP+TN}{TP+TN+FP+FN}$$

(4) $$\mathrm{Sensitivity}=\;\frac{TP}{TP+FN}$$

(5) $$\mathrm{Specificity}=\;\frac{TN}{TN+FP}$$

(6) $$F1=\;\frac{2TP}{2TP+FP+FN}$$

(7) $$\mathrm{MCC}=\;\frac{TP\;\times \;TN-FP\;\times \;FN}{\sqrt{\left(TP+FP)(TP+FN)(TN+FP)(TN+FN\right)}}$$

Here, true positive (TP), true negative (TN), false positive (FP), and false negative (FN) are calculated for the above performance metrics. In the above equations, sensitivity measures the model’s ability to predict each category’s true positives, and specificity measures the model’s ability to predict true negatives. Further, Mathew’s correlation coefficient (MCC) measures the correlation between predicted and actual labels, and F scores measure model accuracy. They combine the model’s precision and recall scores. The receiver operating characteristic curve (ROC) is a probability curve, and the area under the curve (AUC) measures separability between two labels.

### 2.6 Evaluation of model performance on an independent dataset

To evaluate the model’s generalization capability, an independent test dataset was curated from published studies on DNA damage response in human cells (Zimmermann *et al*. 2018, Hustedt *et al*. 2019). To avoid any data leakage, the test set was filtered by gene identifiers and symbols to ensure unique gene entries that were not included in the training dataset. Node2vec was then applied to obtain embeddings for this test dataset using the same criteria as the training set. The resulting test dataset comprised of 312 DDR genes and 8266 non-DDR genes. Given the larger number of non-DDR genes compared to those in the training data, an equal number were randomly selected for testing purposes. Model evaluation on this test dataset was repeated five times to ensure reliable results by taking their average performance.

We also used the models to classify the protein-coding genes in the GENCODE dataset in order to evaluate GlncDDR’s ability to identify additional DDR-associated genes. First, all protein-coding genes annotated in GENCODE Release 36 (GRCh38.p13) were retrieved and matched to their corresponding expression profiles from the TCGA pan-cancer dataset. Genes present in the original training and test datasets were removed to ensure unbiased assessment. For the remaining genes, co-expression graphs were constructed using WGCNA, followed by node2vec-based embedding using the same parameters as in model training. The resulting embeddings were then fed into the trained models (LR, RF, and SVM) to generate DDR-association scores. Genes predicted as DDR-associated by at least two out of three models were considered high-confidence candidates. For downstream interpretation, the predicted genes were compared post hoc to published DDR gene sets from prior experimental studies (Olivieri *et al*. 2020).

### 2.7 Prediction of candidate lncRNAs associated with DDR

After cross-validation and testing, GlncDDR models were used to predict candidate lncRNAs associated with DDR. The lncRNAs were sourced from GENCODE Release 36 (https://www.gencodegenes.org/human/release_36.html). A total of 40,683 lncRNAs were compiled after the filtering and preprocessing steps as performed for the training and test datasets. After performing the prediction, we designed a strategy to prioritize the candidate lncRNAs for future experimental validation. We considered the lncRNAs predicted by all three learning algorithms as the candidate lncRNAs. Furthermore, we performed some analyses to examine the candidate lncRNAs. We searched the literature to identify any existing knowledge connecting the candidate lncRNAs with DDR. We also analyzed genomic location proximity to examine the possible functional similarity and shared regulatory interactions between the candidate lncRNAs and protein-coding DDR genes. By employing a combination of prediction, annotation, literature search, and genomic location proximity analysis, we aimed to identify a high-confidence set of candidate lncRNAs with a strong likelihood of involvement in DDR.

## 3 Results and discussion

### 3.1 Prediction of DDR genes using cancer transcriptomic data

**Figure 2:**
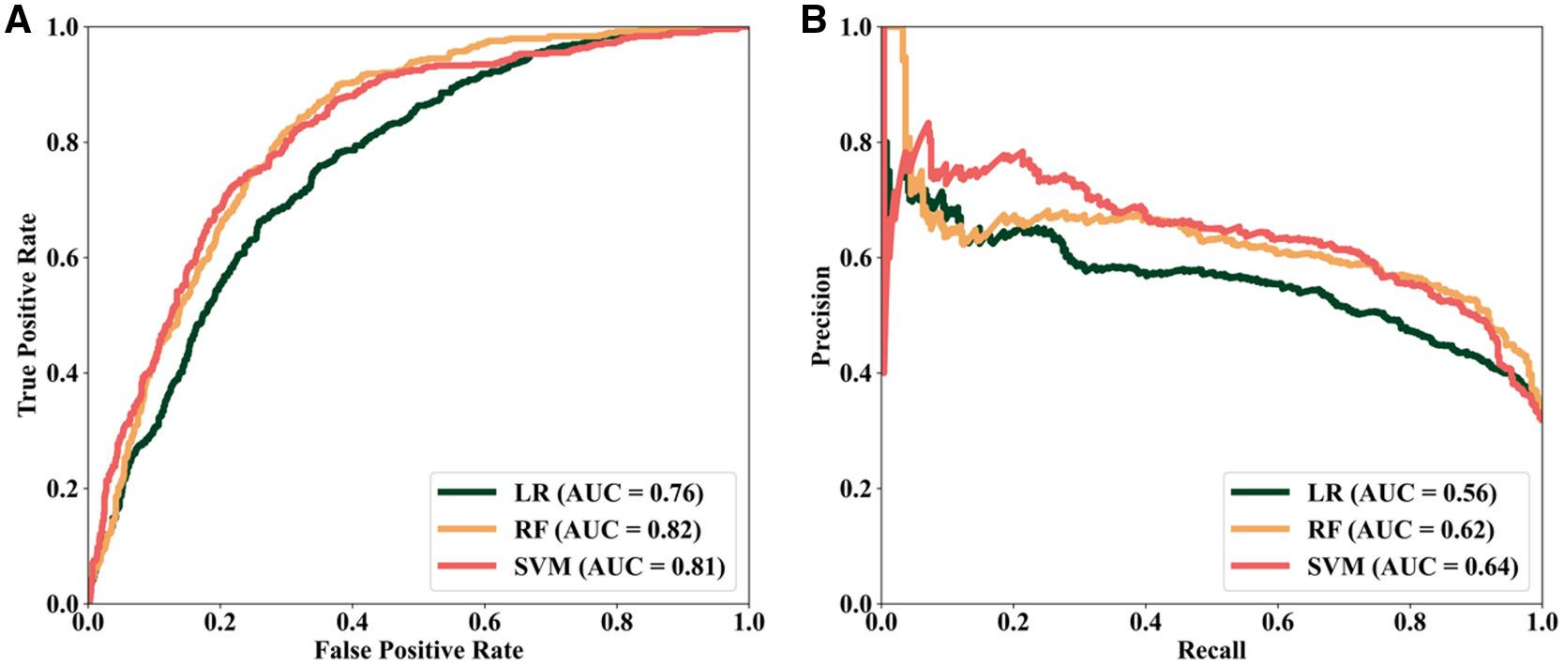
Model performance using full TCGA expression features. (A) Receiver Operating Characteristic (ROC) curve for each machine learning algorithm (LR, RF, or SVM) based on five repetitions of fivefold cross-validation. A higher ROC-AUC value indicates a better model capability to distinguish between DDR and non-DDR genes. (B) Precision Recall (PR) curves to show model effectiveness in identifying true positives while minimizing false positives.

To predict DDR-associated lncRNAs, we utilized the TCGA dataset, a comprehensive collection of transcriptomic data from various cancer types. TCGA is a valuable resource for investigating the expression patterns and pathways of DDR genes in cancer cells. To construct models for DDR gene prediction, we initially used all the expression features (11,273 tumor samples) from the TCGA dataset, and model performance was evaluated using five-fold cross-validation. As shown in Fig. 2, the RF and SVM models showed relatively high performance with ROC-AUC at 0.82, followed by the LR model at 0.76. While the model performance, as indicated by the ROC-AUC values, is promising, the high dimensionality of the TCGA transcriptomic data may result in susceptibility to model overfitting (Supplementary Table S1, available as supplementary data at *Bioinformatics Advances* online). Thus, dimensionality reduction techniques could help mitigate the overfitting issue and improve model generalization capabilities.

**Figure 3:**
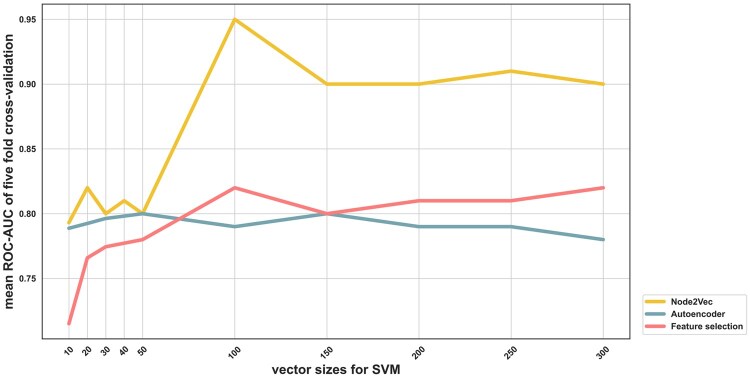
Effects of feature selection and representation learning on model performance. The SVM models with expression features of various dimensionality sizes (10, 20, 30, 50, 150, 200, 250, and 300) were assessed using ROC-AUC. Average ROC-AUC was calculated from five repetitions of five-fold cross-validation.

To examine the effect of dimensionality reduction on model performance, we employed a conventional RF-based feature selection approach to eliminate superfluous and extraneous features. The RF algorithm assigns importance scores to features based on their effectiveness in reducing decision tree impurity. The top-ranked features identified by the RF algorithm were then used for model training and validation. The average ROC-AUC values based on five-fold cross-validation showed some variations across different feature set sizes, ranging from 0.72–0.80 for LR, 0.75–0.84 for RF, and 0.72–0.82 for SVM (Fig. 3, and Supplementary Fig. S1, available as supplementary data at *Bioinformatics Advances* online). Nevertheless, we did not observe a significant improvement in model performance through feature selection. The results suggest that while the conventional RF-based feature selection method effectively reduces dimensionality, it may not adequately capture the intricate and potentially non-linear relationships in the transcriptomic data.

### 3.2 Feature representation learning to improve model performance

Since the conventional feature selection approach did not improve model performance, we next tested deep learning-based feature representation learning with autoencoders. Autoencoders are neural networks that compress high-dimensional input into a latent space and reconstruct the original input, enabling them to learn nonlinear structures in the data. We implemented a classical deep autoencoder (encoder: 11,273 → *d*, decoder: *d* → 11,273) and trained it to reconstruct transcriptomic profiles. The resulting latent features were subsequently used for model training with different machine learning algorithms (LR, RF, and SVM). As shown in Fig. 3 and Supplementary Fig. S1, available as supplementary data at *Bioinformatics Advances* online, model performance using autoencoder-derived features was comparable to that of models trained on the full feature set or RF-selected feature subsets. The ROC-AUC values ranged from 0.75 to 0.80 for LR, 0.80–0.81 for RF, and 0.78–0.80 for SVM. While the autoencoder effectively reconstructed the gene expression data, its latent features did not enhance model performance, suggesting that nonlinear compression alone was insufficient to capture discriminative information for DDR gene classification.

Next, we investigated whether incorporating inter-gene relationships, inferred from expression similarity, could improve model performance. DDR is a complex process, orchestrated by interactive pathways involving damage recognition, signal transduction, and DNA repair. These processes are modular, interdependent, and frequently involve feedback loops and co-regulated components. Capturing such biological coordination may require models representing gene activity and the structural context in which genes function. We thus constructed an undirected gene graph, in which nodes represent genes and edges model pairwise similarity in expression across samples. Node2vec, a graph embedding algorithm, was used to learn low-dimensional representations of nodes by simulating biased random walks through the graph. This method captures local and global topological features, encoding co-expression patterns and the structural roles of each gene within the graph. Compared to feature selection and autoencoder approaches, node2vec offers the advantage of preserving gene contextual proximity and neighborhood structure, which align well with DDR pathways. Moreover, node2vec captures the structural context of individual nodes through flexible, biased random walks, and these walks can be tuned to emphasize community-based proximity or structural equivalence, enabling the method to encode each gene based on its relational profile within the expression-derived graph. This capacity to generate discriminative, context-aware embeddings without requiring labeled nodes makes node2vec particularly well-suited for gene-level prediction tasks, where the objective is to derive informative representations from high-dimensional, unstructured transcriptomic data. Notably, node2vec-derived embeddings were shown to significantly improve model performance; ROC-AUC values ranged from 0.84 to 0.95 for LR, 0.87–0.95 for RF, and 0.79–0.95 for SVM, with various embedding dimensions (Fig. 3, and Supplementary Fig. S1, available as supplementary data at *Bioinformatics Advances* online). Moreover, the model performance improvement appeared to be driven by certain embedding dimensions (Supplementary Fig. S2, available as supplementary data at *Bioinformatics Advances* online). Although the biological interpretation of individual embedding dimensions remains limited, the results suggest that graph‐informed embeddings may capture the relevant network context needed to distinguish DDR genes.

**Figure 4:**
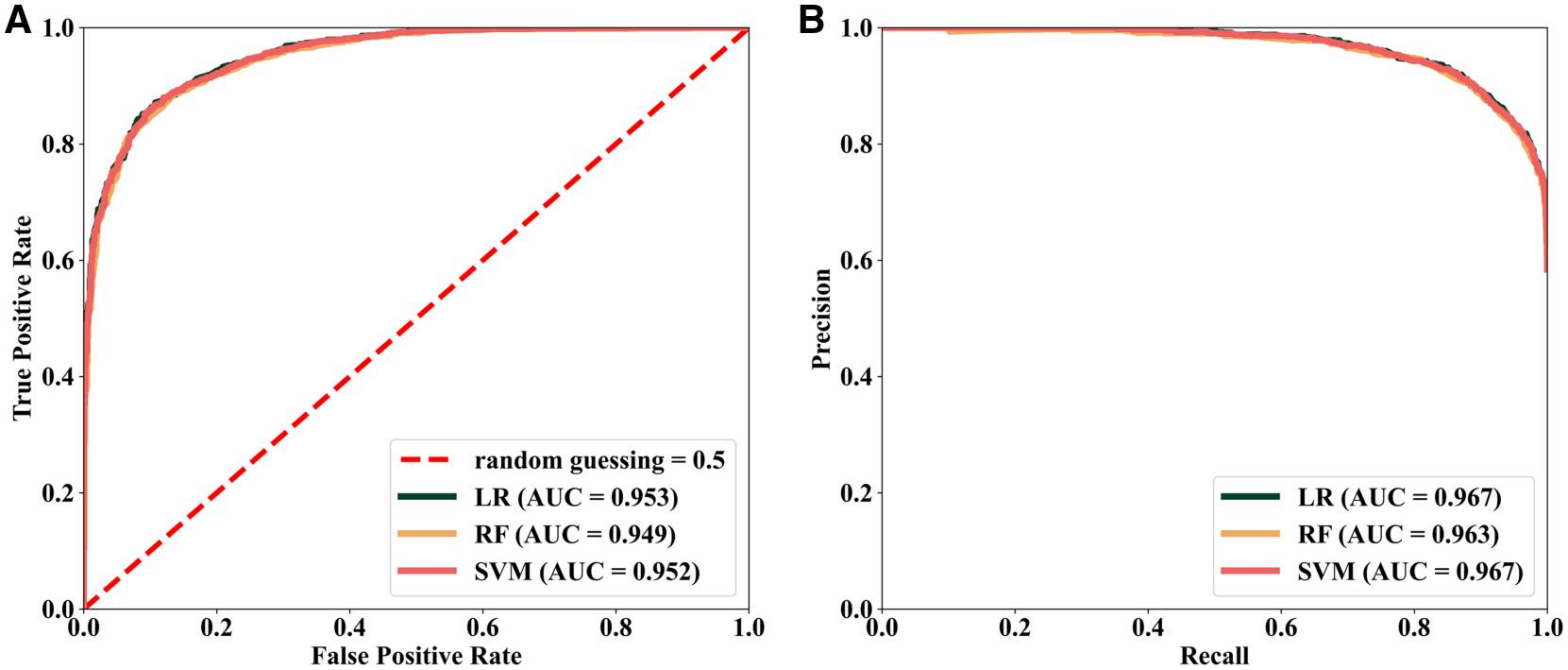
Predictive performance of GlncDDR models. (A) ROC curves of LR, RF and SVM models based on five-fold cross-validation. (B) PR curves of the models for predicting DDR genes.

We further examined the performance of LR, RF, and SVM models over a range of embedding dimensions (Fig. 3, Supplementary Fig. S1, and Supplementary Table S2, available as supplementary data at *Bioinformatics Advances* online) to determine the optimal feature vector size. Model performance improved with increasing vector sizes, and peaked at the vector size of 100, where all three models achieved an ROC-AUC of ∼0.95. The results agree with a previous network representation learning study (Grover and Leskovec 2016), in which model performance was found to generally plateau once embedding dimensionality reached 100. With this optimal feature vector size, GlncDDR models achieved superior performance as indicated by ROC-AUC (∼0.95), accuracy (0.82–0.86), sensitivity (0.72–0.81), specificity (0.94–0.97), MCC (0.68–0.73), and F1 score (0.83–0.87) (Table 1). Figure 4 shows the ROC and PR curves of the LR, RF, and SVM models. The consistency of superior performance across different models further demonstrates the effectiveness of the graph-based feature representation learning approach.

**Table 1:** Performance metrics of GlncDDR models based on five-fold cross-validation.

Model	Accuracy	Sensitivity	Specificity	MCC	F1 Score	ROC-AUC
LR	0.85	0.79	0.94	0.72	0.86	0.95
RF	0.86	0.81	0.94	0.73	0.87	0.95
SVM	0.82	0.72	0.97	0.68	0.83	0.95

### 3.3 Robust performance of GlncDDR on independent test data

**Figure 5:**
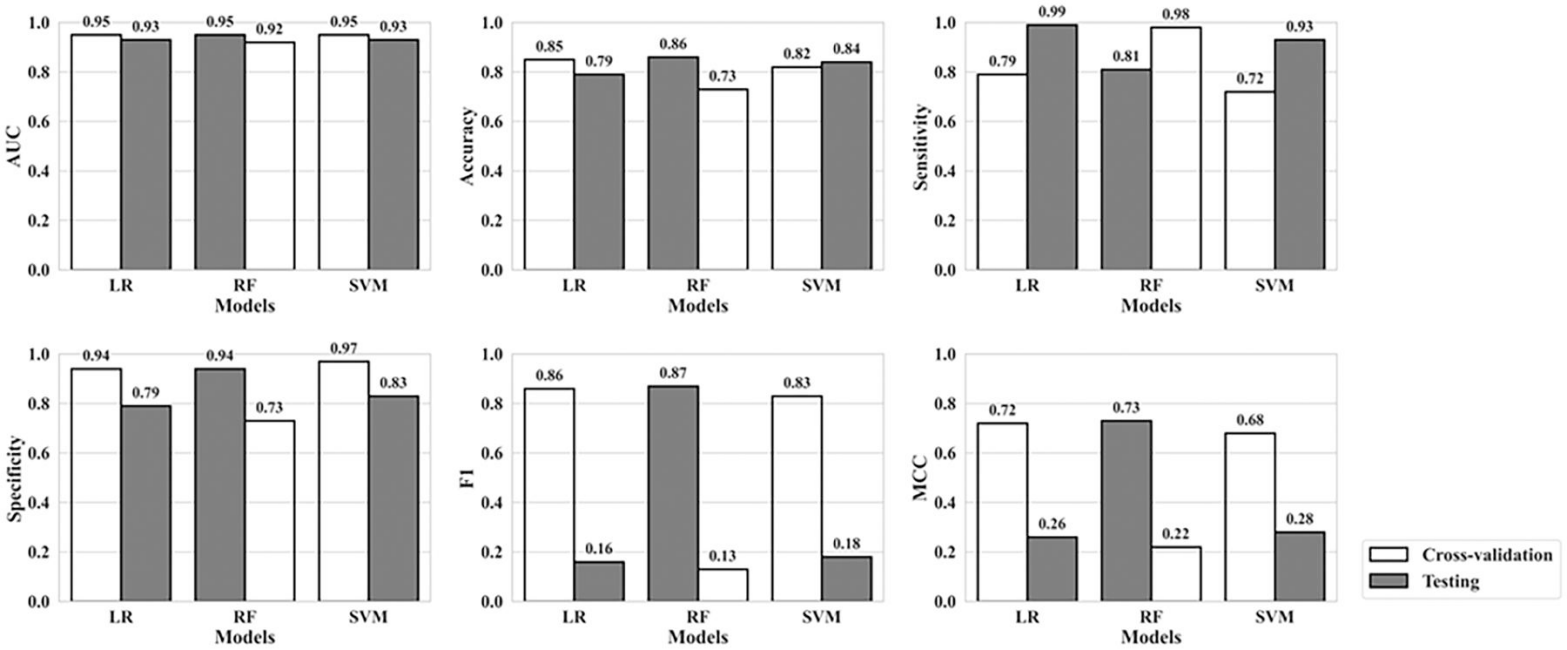
Comparison of model performance in independent testing and cross-validation. GlncDDR models were evaluated using an independent test dataset. The peach bars indicated model performance on the test data, whereas the green bars indicate model performance in cross-validation with the training data.

To evaluate the generalizability of GlncDDR models, we compiled an independent test dataset consisting of 161 DDR-associated protein-coding genes and 905 non-DDR genes. None of the test instances overlapped with the training data. This test dataset was processed using the same pipeline applied to the training dataset, with node2vec embedding dimensionality of 100. As shown in Figure 5, GlncDDR models achieved high ROC-AUC (0.93 for LR, 0.92 for RF, and 0.93 for SVM), comparable with the performance during training. Test accuracy ranged from 73% to 84%, with SVM achieving the highest accuracy. The models achieved higher sensitivity (0.93–0.99), but lower specificity (0.73–0.83). The F1 and MCC performance metrics also decreased, indicating the difficulty in correctly predicting the minority-class DDR genes in the highly imbalanced dataset. Similar results were obtained using the class-weight and threshold optimization methods for the minority-class prediction (Supplementary Table S3, available as supplementary data at *Bioinformatics Advances* online). Nevertheless, the overall test results suggest that GlncDDR models can maintain robust predictive performance on unseen data.

To further evaluate GlncDDR’s capability to identify DDR-associated genes, we used the models to classify the protein-coding genes in the dataset of GENCODE Release 36. After excluding all the genes in the training and test datasets, GlncDDR predicted 291 additional protein-coding genes potentially associated with DDR (Supplementary Table S4, available as supplementary data at *Bioinformatics Advances* online). Notably, 133 or ∼46% of the predicted DDR genes were also reported in a recent experimental study (Olivieri *et al*. 2020) as DDR genes, including *SAMD4B, GPRC5D, ARPC4, CENPO, CCDC6, XRN1, VARS2, SNRPB2, OSTC, IRX6, CDK12*, and *IL6ST*. These findings provide additional evidence for the high performance of GlncDDR to predict novel DDR-associated genes based on expression patterns in cancer cells.

### 3.4 Genome-wide identification of candidate lncRNAs associated with DDR

After demonstrating GlncDDR’s predictive capability, we next used the models to predict DDR-associated lncRNAs in the GENCODE dataset (Release 36). To minimize false-positive predictions and prioritize high-confidence candidate lncRNAs, we employed a multi-level filtering strategy. Firstly, we implemented a stringent consensus filter, retaining only those lncRNAs predicted by all three models with a probability ≥ 0.6 (Supplementary Table S5, available as supplementary data at *Bioinformatics Advances* online) to identify high-confidence candidate lncRNAs as putative novel DDR regulators. To assess the potential regulatory roles of these candidate lncRNAs, we examined their genomic overlap or proximity with known DDR genes, which served as positive examples for model training. LncRNAs often exert *cis*-regulatory effects on nearby protein-coding genes by influencing their transcription or altering chromatin structure (Statello *et al*. 2021). GlncDDR identified 85 candidate lncRNAs overlapping with the DDR genes (Supplementary Table S6, available as supplementary data at *Bioinformatics Advances* online), and 132 lncRNAs in proximity to the DDR genes (within 50 kb upstream and downstream) (Supplementary Table S7, available as supplementary data at *Bioinformatics Advances* online), suggesting that these lncRNAs might be involved in DDR pathways through *cis*-regulatory mechanisms. We further analyzed the candidate lncRNAs using Genomic Regions Enrichment of Annotations Tool (GREAT) to assess the functional significance of *cis*-regulatory regions (McLean *et al*. 2010). Notably, the candidate lncRNA loci show significant enrichment for functional annotations related to canonical DDR pathways. Functional terms associated with the ATM/ATR signaling network, p53 target genes, cell-cycle checkpoints, and DNA repair processes are among the top of the enrichment list (Supplementary Table S8, available as supplementary data at *Bioinformatics Advances* online). The results suggest that many candidate lncRNA genes reside in the genomic regions highly pertinent to DNA damage sensing and repair.

Importantly, the candidate lncRNAs predicted by GlncDDR (Supplementary Table S5, available as supplementary data at *Bioinformatics Advances* online) also include many known to be involved in DDR, including *JADRR, PINCR, TP53TG1, HOTAIR, MALAT1, ENRICD*, and *DINOL*. *JADRR*, also known as *lncRNA-JADE*, links ATM-dependent DNA damage signaling to chromatin modification by promoting histone H4 acetylation (Wan *et al*. 2013). *PINCR* is a p53-inducible lncRNA that is strongly upregulated upon DNA damage and regulates a subset of p53 target genes to enforce G1 arrest and cell survival (Chaudhary *et al*. 2017). *TP53TG1* (TP53 Target Gene 1) is another p53-responsive lncRNA required for an optimal p53-mediated DDR, acting as a tumor-suppressive regulator of p53 signaling (Cheng *et al*. 2022). *HOTAIR*, a well-known chromatin-modifying lncRNA, facilitates DNA repair signaling through interacting with the ATR kinase and enhancing the expression of DNA repair factors (Özeş *et al*. 2016). *MALAT1*, an abundant nuclear lncRNA, influences DDR by modulating gene expression and repair pathways, and its overexpression enhances the DNA repair capacity of cells and confers resistance to genotoxic stress (Hou *et al*. 2023). Finally, *DINOL* (Damage-Induced Noncoding RNA) is a p53-activated lncRNA that stabilizes the p53 protein, creating a positive feedback loop that amplifies DNA damage signaling and promotes proper cell-cycle arrest and apoptosis (Schmitt *et al*. 2016). The positive predictions by GlncDDR for these known DDR-associated lncRNAs underscore the model’s predictive power for identifying novel candidates.

Of the 1232 novel candidate lncRNAs predicted by GlncDDR, seven are highlighted in Table 2 to suggest their possible involvement in DDR. Four of the candidates (*ALKBH3-AS1, IRF1-AS1, POT1-AS1*, and *WAC-AS1*) are antisense or proximal to known DDR genes and thus may play *cis*-regulatory roles. *ALKBH3-AS1* is transcribed antisense to *ALKBH3*, which encodes DNA alkylation repair enzyme. Although *ALKBH3-AS1* has not been explicitly characterized as a DDR-associated lncRNA, its possible involvement in DDR is supported by several observations. It is overexpressed in hepatocellular carcinoma and enhances *ALKBH3* mRNA stability (Lu *et al*. 2022). Moreover, *ALKBH3-AS1* expression is transcriptionally activated by HIF-1α under the condition of hypoxia, which is known to suppress multiple DNA repair pathways, including mismatch repair and homologous recombination (Lu *et al*. 2022). *IRF1-AS1* is associated with prognostic m6A-related lncRNAs responsible for causing cancer, neurological disorders, and immune system dysfunctions (Zhao *et al*. 2021). Furthermore, *IRF1-AS1*, along with *MCM3AP-AS1 and TRAF3IP2-AS1*, is part of a subnetwork linked to genes involved in DNA repair (Nekoeian *et al*. 2022). These lncRNAs may sponge *Has-miR-144*, a microRNA that regulates cell proliferation and apoptosis in acute lymphoblastic leukemia (Jin *et al*. 2017). Thus, *IRF1-AS1* is involved in cancer-related pathways and may play a regulatory role in DDR. *POT1-AS1* overlaps the telomere-protecting gene *POT1* (Wu *et al*. 2006)*. POT1-AS1* is known to regulate telomere maintenance, a critical aspect of genome stability. As disruptions in telomere maintenance are linked to increased sensitivity to DNA damage, *POT1-AS1* can be an interesting target for further experimental investigations. *WAC-AS1* is an oncogenic lncRNA involved in tumor progression and stress-response regulation (Liu *et al* 2024). *WAC-AS1* is antisense to *WAC*, which partners with the RNF20/40 E3 ligase complex to mediate histone H2B monoubiquitination and is required for DNA-damage-induced checkpoint activation and transcriptional responses to genomic stress (Zhang and Yu 2011).

**Table 2:** Selected candidate lncRNAs predicted by GlncDDR.

Candidate lncRNA	Neighboring genes	LncRNA biotype	Functional description	References
ALKBH3-AS1	ALKBH3	Antisense	Regulates DDR through post-transcriptional stabilization of ALKBH3, resulting in elevated protein levels that catalyze oxidative demethylation of alkylated DNA bases.	Lu *et al.* 2022
LINC02381	SMUG1, CBX5	Intergenic	Binds to transcription factor CEBPβ at CBX5, increasing transcription of CBX5 which is known to influence DNA repair.	Naqvi *et al.* 2023
POT1-AS1	—	Antisense	Competing endogenous RNA—sponges miR-497-5p, elevating PDK3 expression and promoting survival under genotoxic stress (chemo-resistance).	Chen *et al.* 2021
LINC00963	—	Intergenic	Promotes radioresistance and DNA‐damage tolerance by sponging miR-324-3p/ACK1 and activating FOSB/UBE3C-mediated TP73 degradation, thereby reducing γH2AX foci and overriding cell-cycle checkpoints.	Zhang *et al.* 2019; Wang *et al* 2023
WAC-AS1	WAC	Antisense	WAC-AS1 levels positively correlated with DNA repair signatures in certain cancers.	Wang *et al.* 2023
IRF1-AS1	—	Antisense	Functions as a tumor suppressor by regulating cell proliferation and apoptosis; IRF1 regulates DNA damage-induced apoptosis and cell cycle arrest.	Jin *et al* 2017
C8orf86	EYA1, PRKDC, RAD21, TCEA1, PINX1	Intergenic	A direct p53 transcriptional target; knockdown of C8orf86 induces tumor growth by reducing p53 transcriptional activity.	Zhang *et al* 2023

Interestingly, three candidate lncRNAs, *C8orf86, LINC0096*3, and *LINC02381*, are intergenic transcripts not previously shown to be directly involved in DDR. Particularly, *C8orf86 (LINC00324)*, a long intergenic lncRNA linked to periampullary adenocarcinoma, has been shown to modulate transcriptional regulation by p53, a key regulator of DDR (Vousden and Prives 2009). *C8orf86* may enhance the transcriptional activity of p53 by interfering with the p53-SET interaction, which leads to increased acetylation of p53 target promoters and upregulation of p53-dependent genes (Wang *et al*. 2016). The knockdown of *C8orf86* has been shown to reduce p53 transcriptional activity, leading to enhanced tumor growth (Zhang *et al*. 2023). However, further investigations are needed to understand the direct role of *C8orf86* in DDR. *LINC00963* has been reported as an oncogenic lncRNA in cancer, and it may function as a microRNA sponge or by interacting with DDR-related proteins. *LINC02381* is an intergenic lncRNA, which may influence DDR gene expression through chromatin regulation. These novel candidate lncRNAs predicted by GlncDDR merit further experimental studies to confirm their roles in DDR and to understand the molecular mechanisms.

## 4 Conclusion

We have developed a new machine learning approach, GlncDDR, to predict candidate lncRNAs associated with DDR using cancer transcriptomic data and graph neural networks for feature representation learning. The high predictive performance of GlncDDR on independent test data demonstrates its robustness and reliability. Thus, GlncDDR provides a valuable alternative to experimental methods for identifying novel DDR-associated genes. As lncRNAs are increasingly recognized for their roles in cancer and stress responses, the candidates predicted by GlncDDR can serve as promising new avenues for DDR research. Further characterization of these candidate lncRNAs could not only deepen our understanding of DDR pathways but also potentially unveil novel biomarkers or therapeutic targets for diseases caused by DNA damage dysregulation. While GlncDDR achieved superior performance in the present study, model robustness and prediction reliability may be further enhanced by examining other graph-based approaches and ensemble integration, as well as sampling and cost-sensitive learning methods to better address class imbalance within the training and test datasets.

## Supplementary Material

vbag119_Supplementary_Data
