# Supplementary material for: Accurate prediction of candidate lncRNAs associated with DNA damage response based on gene expression patterns from graph neural networks
Source: Bioinform Adv. 2026 Apr 26;6(1):vbag119. doi: 10.1093/bioadv/vbag119 (PMC13143433; doi:10.1093/bioadv/vbag119)
Supplement: vbag119_Supplementary_Data [file vbag119_supplementary_data.zip › Supplementary_Information.docx]

**Supplementary information for**

**Accurate prediction of candidate lncRNAs associated with DNA damage response based on gene expression patterns from graph neural networks**

Snehal Shah^1,2,*^ and Liangjiang Wang^1,2,*^

^1^Department of Genetics and Biochemistry, Clemson University, Clemson, SC 29631, USA

^2^Centre for Human Genetics, Clemson University, Greenwood, SC 29646, USA

^*^To whom correspondence should be addressed, liangjw@clemson.edu or snehals@clemson.edu

**Supplementary Figure S1. Performance comparison of Logistic Regression (LR) and Random Forest (RF) models using different feature selection and representation learning methods.** RF-based feature selection and two feature representation learning methods, including autoencoder and node embedding (node2vec), were used to construct LR and RF models. The average ROC-AUC (area under the curve), calculated from five repetitions of five-fold cross-validation, was used to evaluate the model performance across six different feature vector sizes (10, 20, 30, 40, 50, 100, 150, 200, 250, and 300).

**
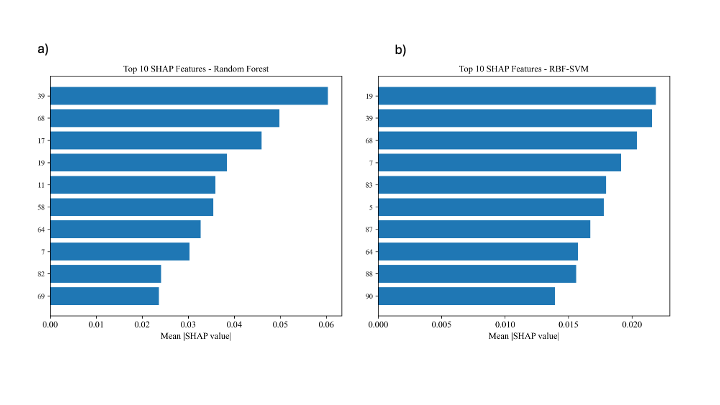
**

**Supplementary Figure S2. Global SHAP feature importance for classifiers trained with node2vec embeddings.** a) Random forest. b) Support vector machine. Embedding dimensions are ranked by their mean absolute SHAP value across test samples. Shap (Shapely Additive exPlanation) quantifies the contribution of each embedding dimension to the predicted probability of the positive class. Mean absolute SHAP values represent the average magnitude of contribution, irrespective of direction, and therefore provide a measure of global feature importance. Higher values indicate greater overall influence on model prediction. In both classifiers, a limited subset of embedding dimensions accounts for most of the predictive contribution, while most dimensions have comparatively low impact. Notably, several top-ranked dimensions (e.g., 39, 68, 19, 7, and 64) are shared between RF and SVM models despite their distinct learning mechanisms. This cross-model consistency suggests that the predictive signal is concentrated in specific components of the learned embedding representation rather than distributed randomly across all features.

**Supplementary Table S1. Performance of models using the full TCGA expression set based on ten-fold cross-validation.**

| Model | Accuracy | Sensitivity | Specificity | MCC | F1 score | ROC-AUC |
| --- | --- | --- | --- | --- | --- | --- |
| LR | 0.712 | 0.582 | 0.773 | 0.349 | 0.561 | 0.762 |
| RF | 0.742 | 0.776 | 0.727 | 0.473 | 0.655 | 0.826 |
| SVM | 0.758 | 0.563 | 0.848 | 0.424 | 0.593 | 0.817 |

**Supplementary Table S2. Model performance across different node embedding dimensions.**

| Model | Node embeddings | | Accuracy | Sensitivity | Specificity | MCC | F1 score | ROC-AUC |
| --- | --- | --- | --- | --- | --- | --- | --- | --- |
| LR | | 10 | 0.70 | 0.12 | 0.98 | 0.19 | 0.20 | 0.84 |
| RF | | 10 | 0.79 | 0.52 | 0.92 | 0.48 | 0.60 | 0.87 |
| SVM | | 10 | 0.77 | 0.57 | 0.87 | 0.46 | 0.61 | 0.79 |
| LR | | 20 | 0.72 | 0.17 | 0.97 | 0.26 | 0.28 | 0.87 |
| RF | | 20 | 0.79 | 0.53 | 0.91 | 0.49 | 0.62 | 0.88 |
| SVM | | 20 | 0.77 | 0.54 | 0.88 | 0.45 | 0.60 | 0.82 |
| LR | | 30 | 0.72 | 0.18 | 0.98 | 0.29 | 0.30 | 0.88 |
| RF | | 30 | 0.80 | 0.54 | 0.92 | 0.50 | 0.62 | 0.88 |
| SVM | | 30 | 0.78 | 0.49 | 0.91 | 0.45 | 0.58 | 0.80 |
| LR | | 40 | 0.74 | 0.26 | 0.96 | 0.34 | 0.38 | 0.89 |
| RF | | 40 | 0.82 | 0.62 | 0.92 | 0.58 | 0.69 | 0.90 |
| SVM | | 40 | 0.78 | 0.57 | 0.88 | 0.48 | 0.63 | 0.81 |
| LR | | 50 | 0.74 | 0.27 | 0.97 | 0.35 | 0.40 | 0.90 |
| RF | | 50 | 0.81 | 0.59 | 0.91 | 0.53 | 0.66 | 0.90 |
| SVM | | 50 | 0.79 | 0.57 | 0.89 | 0.49 | 0.63 | 0.80 |
| LR | | 100 | 0.85 | 0.79 | 0.94 | 0.72 | 0.86 | 0.95 |
| RF | | 100 | 0.86 | 0.81 | 0.94 | 0.73 | 0.87 | 0.95 |
| SVM | | 100 | 0.82 | 0.72 | 0.97 | 0.68 | 0.83 | 0.95 |
| LR | | 150 | 0.78 | 0.96 | 0.71 | 0.62 | 0.74 | 0.9 |
| RF | | 150 | 0.78 | 0.94 | 0.71 | 0.61 | 0.73 | 0.88 |
| SVM | | 150 | 0.74 | 0.99 | 0.62 | 0.59 | 0.71 | 0.9 |
| LR | | 200 | 0.77 | 0.36 | 0.96 | 0.42 | 0.49 | 0.91 |
| RF | | 200 | 0.81 | 0.66 | 0.88 | 0.56 | 0.69 | 0.90 |
| SVM | | 200 | 0.79 | 0.62 | 0.87 | 0.51 | 0.65 | 0.83 |
| LR | | 250 | 0.79 | 0.96 | 0.71 | 0.63 | 0.75 | 0.90 |
| RF | | 250 | 0.78 | 0.94 | 0.71 | 0.60 | 0.73 | 0.88 |
| SVM | | 250 | 0.75 | 1.00 | 0.63 | 0.59 | 0.72 | 0.91 |
| LR | | 300 | 0.79 | 0.95 | 0.71 | 0.62 | 0.74 | 0.90 |
| RF | | 300 | 0.78 | 0.93 | 0.71 | 0.60 | 0.73 | 0.88 |
| SVM | | 300 | 0.74 | 1.00 | 0.62 | 0.59 | 0.71 | 0.90 |

**Supplementary Table S3. Performance of GlncDDR on independent test data using F2-optimized thresholds.**

| Model | Accuracy | Sensitivity | Specificity | MCC | F1 score | ROC-AUC |
| --- | --- | --- | --- | --- | --- | --- |
| LR | 0.789 | 0.971 | 0.785 | 0.250 | 0.153 | 0.923 |
| RF | 0.680 | 0.100 | 0.674 | 0.198 | 0.110 | 0.870 |
| SVM | 0.790 | 0.971 | 0.787 | 0.251 | 0.154 | 0.921 |
